# ‘Popping nana back into bed’ - a qualitative exploration of paramedic decision making when caring for older people who have fallen

**DOI:** 10.1186/s12913-017-2243-y

**Published:** 2017-04-21

**Authors:** Paul Simpson, Ric Thomas, Jason Bendall, Bill Lord, Stephen Lord, Jacqueline Close

**Affiliations:** 1 Western Sydney University, Locked Bag 1797, Penrith, 2750 NSW Australia; 2 University of Sunshine Coast, Queensland, Australia; 3 Falls, Balance and Injury Research Centre, Neuroscience Research Australia, University of New South Wales, Sydney, NSW Australia; 4 Department of Geriatric Medicine, Prince of Wales Clinical School, Sydney, Australia

**Keywords:** Paramedic, Decision making, Older, Falls

## Abstract

**Background:**

Older fallers constitute a large proportion of ambulance work, and as many as 25% are not transported to hospital following paramedic assessment. The objective of this study was to explore the decision making process used by paramedics when caring for older fallers.

**Methods:**

A qualitative study was conducted using constructivist grounded theory methodology. Purposive sampling was used to recruit paramedics to participate in semi-structured interviews and focus groups. Data analysis commenced with line-by-line coding, developing into formation of theoretical categories. Theoretical sampling was then used to clarify emerging theoretical concepts, with data collection and analysis continuing until theoretical saturation was achieved.

**Results:**

A total of 33 paramedics participated in 13 interviews and 4 focus groups. When caring for older fallers, paramedic decision making is profoundly affected by ‘role perception’, in which the individual paramedic’s perception of what the role of a paramedic is determines the nature of the decision making process. Transport decisions are heavily influenced by a sense of ‘personal protection’, or their confidence in the ambulance service supporting their decisions. ‘Education and training’ impacts on decision making capacity, and the nature of that training subliminally contributes to role perception. Role perception influences the sense of legitimacy a paramedic attaches to cases involving older fallers, impacting on patient assessment routines and the quality of subsequent decisions.

**Conclusions:**

Paramedic decision making processes when caring for older people who have fallen appear to be strongly influenced by their perception of what their role should be, and the perceived legitimacy of incidents involving older fallers as constituting ‘real’ paramedic work.

## Background

While once largely a protocol-driven vocation with little autonomy and limited clinical responsibility, evolving models of contemporary paramedicine involve broad scopes of practice and demand increased levels of clinical decision making, judgement and professional accountability. The changing demography of healthcare consumers accessing paramedic resources [1] together with an increased focus on models of care aimed at providing alternatives to emergency department (ED) care for patients with perceived low acuity presentations, call for a higher level of clinical decision making and clinical judgement than that which was historically required.

Paramedic practice involves high-density decision making [2], with a high volume of decisions necessary in a relatively constrained timeframe. Paramedics are not only required to make clinical decisions relating to assessment, diagnosis, immediate management and disposition; they are also required to make multiple operational, logistic and resource-management decisions. These decisions are commonly made under less than ideal circumstances, with only limited diagnostic tests and investigations available and relatively short time frames in which to build a rapport with the patient, elicit important history, examine the patient, synthesise findings and implement required treatments.

While delivery of timely care to patients with acute presentations remains a fundamental part of service delivery, paramedics are increasingly attending patients with less urgent and less acute conditions [2–4]. A proportion of these patients may benefit from not being transported to an ED, instead being discharged at the scene and streamlined into community-based services provided by primary or allied health agencies [5, 6]. The decision regarding the most appropriate disposition for such patients is complex and requires sound clinical reasoning and judgement in order to provide safe and effective care.

Older people who have fallen from a standing height account for 6–8% of ambulance service annual emergency work load [7–9]. Between 25 and 40% of these responses result in the patient not being transported to an emergency department (ED), a proportion substantially greater than that seen in other ambulance patient populations [10]. While commonly perceived to be low acuity, these cases can be complex, requiring paramedics to make sound and insightful clinical decisions, especially with regard to disposition.

Paramedic decision making in cases involving older fallers has been the subject of a previous qualitative study involving a convenience sample of 12 paramedics in the United Kingdom [11]. That study highlighted the difficulties encountered by paramedics when making decisions surrounding the care and management of older fallers. The researchers identified an unstructured, non-systematic decision making process that was in need of supportive processes such as decision algorithms or protocols to guide safe practice. This study also identified a lack of appreciation by participating paramedics of the complexity of the decisions they were making, evidenced by a reluctance to use, and an indifferent attitude towards an available algorithmic falls-decision tool. Other studies have investigated paramedic decision making in other contexts including high acuity care [2, 12], morphine administration [13], post-seizure management [14], activation of catheterisation labs [15], intravenous cannulation [16] and acute mental health presentations [17]. Decision making in less urgent situations has not been investigated despite the increasing volume of this category of work and the complexity that may be involved [18].

Cases that involve non-specific clinical findings require critical analysis to formulate a clinical impression and subsequent management plan. For example, the cause of a fall sustained by an older person may be assessed as a simple trip, resulting in early diagnostic closure and missed opportunity to explore factors that may have caused or contributed to the fall. Failure to recognise errors in decision making such as premature diagnostic closure and overconfidence in the assessment of the need for further medical assessment may compromise patient safety. This might be compounded by a generally incomplete feedback loop regarding patient outcomes that would assist in monitoring of clinical performance. Against this background of growing demand for ambulance services and a desire to identify opportunities for patients to be safely diverted to non-ED alternatives, this qualitative study aimed to investigate paramedic decision making and develop a theory explaining paramedic decision making whilst providing care to older people who have fallen.

## Methods

### Methodology

We employed a qualitative study design incorporating constructivist grounded theory methodology. Grounded theory (GT) research aims to describe and understand processes and actions, and as our aim was to develop a theory explaining how paramedics make decisions about the clinical care of older patients who have fallen, the methodology of GT was appropriate [19]. The GT framework is also well suited when little is already known about the area being researched as is the case with paramedic decision making [19].

In designing and conducting the study, we considered ontological and epistemological positions, as these concepts directly impact the nature of data collection and analysis. Our ontological position was one of relativism, and our epistemological stance was one of subjectivism, underpinning a constructivist theoretical framework for our grounded theory methodology. Originating from the traditional grounded theory methodology espoused by Glaser and Strauss [19], constructivist grounded theory is an interpretive stream of GT that acknowledges that qualitative research cannot be objective, and that knowledge arising from such research is not simply an account of the participant’s experience and data, but rather a co-construction between researcher and participant [20]. Essentially, constructivist GT positions the researcher as an active participant in the research, as opposed to an objective observer [20]. This is important to highlight, as the primary investigator for the study, who conducted the interviews and led the analysis, was an experienced paramedic whose own experiences and knowledge impact on interpretation of participant data.

### Study setting

The study was conducted within a large, state-based Australian ambulance service. The service provides road-based and aeromedical prehospital care to a population of approximately 7.4 million people across the state with a geographic area spanning 804,000 square kilometres. The service responds to 1.2 million incidents per year, of which 74% are classified as ‘emergencies’ at the time of ambulance dispatch [21]. Approximately 14% of the population in this region are aged 65 years or more [22]. There are three clinical tiers within this service: qualified paramedics (QP), intensive care paramedics (ICP), and extended care paramedics (ECP). QPs have completed core training to a level equivalent to advanced life support (ALS) and constitute the majority of the 2,794-strong workforce [23]. ICPs have undertaken additional specialist training in intensive care paramedicine with a clinical focus on serious, life threatening injury or illness. ECPs undertake additional specialist training focussed on management of low acuity, minor injury and medical conditions, with a strong focus on common geriatric syndromes and identifying patients suitable for non-emergency department management. ECPs also receive advanced training in clinical decision making, and risk identification, mitigation and management. Paramedics operate under a protocol-based system largely without medical consultation, while ECPs function more autonomously under broader clinical guidelines.

All patients requesting transport are generally taken to an ED, whilst those who are recommended or offered transport and refuse or decline this service are managed by paramedics at the scene without medical consultation. For patients who have fallen, paramedics are provided with an algorithmic ‘Falls protocol’ to guide them in providing clinical care and support decisions as to whether to transport older people following a fall. Under this protocol, older fallers who meet certain clinical criteria may be offered a non-transport alternative care pathway at the discretion of the attending paramedic.

### Participant recruitment

A pool of potential participants was identified through an expression of interest (EOI) released to all operational paramedics via organisational email. The EOI contained a Participant Information and Consent Form, as well as a detailed overview of the study objectives. Participants were offered re-imbursement for travel costs to and from the interview, and incentive in the form of a $50 gift voucher. To enable initial purposive sampling, paramedics who responded to the EOI were asked to provide demographic details relating to length of service, clinical level, geographic location, education and training pathway (i.e. vocational or tertiary). This purposive sampling approach was used to identify participants from whom the initial data could be collected, ensuring a broad cross-section of participant experience, exposure and background. Following commencement of the initial interviews and data collection, subsequent participant selection transitioned from purposive to theoretical sampling. Theoretical sampling, a key element of the grounded theory process, involves sampling of participants who are best positioned to provide data that will provide clarity to, and build on, the theoretical elements emerging from earlier data collection [20]. The process of theoretical sampling continued until ‘theoretical saturation’ had been reached. Theoretical saturation is said to occur when all threads of the emerging theory are deemed to be understood in relation to their individual meaning and their relationship to the overall theory that has been developed [20, 24, 25].

### Data collection methods

Data collection consisted primarily of one-on-one, semi-structured in-depth interviews and focus groups. The initial data were collected from the face-to-face interviews, with focus groups introduced during the theoretical sampling phase to help clarify emerging theoretical concepts. As the analysis progressed various media, organisational and internet sources were also analysed to explore portrayal of paramedicine following the emergence of ‘perception of role’ as the dominant theoretical construct.

**Fig. 1:**
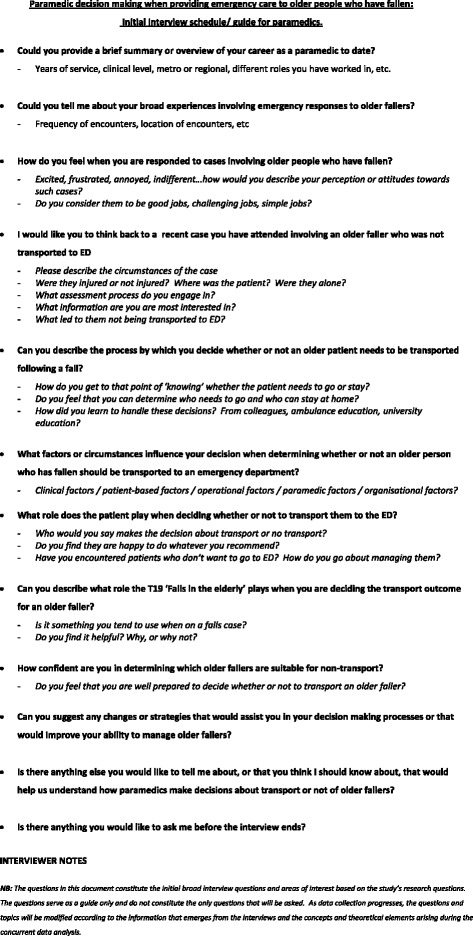
Initial interviewer guide template

All interviews were conducted by the same researcher (PS). The interviews were semi-structured, guided by an initial template that evolved iteratively as the analysis progressed (Fig. 1). In the theoretical sampling phase of data collection, the interview questions focussed primarily on the theoretical concepts that had emerged earlier in the study. Duration of each interview was between 40 and 60 min, conducted over a two month period at mutually agreed locations away from the workplace. The focus groups, consisting of between 4 and 6 paramedics, were facilitated by a researcher experienced in group facilitation (RT), with the primary researcher (PS) present as an observer. Interviews and focus groups were digitally recorded and professionally transcribed in a ‘verbatim’ format. Participant confidentiality was managed by allocating code names to participants prior to the interviews or focus groups taking place. The key to these codes was kept in a password protected database to which only the primary researcher had access. Potentially identifying data within the transcripts were removed by the primary investigator prior to circulation to the research team.

### Data analysis

In keeping with the inductive analytic process underpinning GT methodology, analysis commenced after the first interview and continued concurrent to the ongoing data collection process. Transcripts initially underwent line-by-line open coding, leading to the formation of multiple thematic categories. Axial coding followed, during which the simple categories were analysed and subjected to constant comparison across data sources, leading to the emergence of the theoretical and conceptual categories, which were then analysed to form the categories underpinning the final theoretical model. As data collection was occurring concurrent to analysis, new data were constantly compared to the data that had already been analysed, and new theoretical categories with those that had already emerged. Data were managed using NVivo qualitative data analysis software (QSR International Pty Ltd. Version 10, 2012).

‘Memo-ing’ featured prominently throughout the analysis, and was the primary means by which reflexivity was exercised. Memos were drafted by the interviewer and facilitator immediately following each interview or focus group and conceptual memos were recorded as the analysis process evolved. Interview transcripts, audio files and memos were disseminated amongst the research team and discussed throughout. The potential for researcher bias was addressed through this engagement in reflexive practice, during which personal biases and preconceptions, primarily with regard to the primary researcher conducting the analysis, were identified, declared, and discussed via memos and conversation amongst the research team.

## Results

In total, 33 paramedics participated in the study, of which 12 were female and 21 male. The average length of service was 12 years (SD 6), and 60% were from metropolitan areas. The majority of participants were QPs (*n =* 16), with ICPs (*n =* 11) and ECPs (6). Forty per cent had higher education qualifications in a clinical health discipline.

Initial data collection, consisting of 8 interviews, was conducted using purposive sampling and was accompanied by concurrent data analysis. This was followed by a pause in data collection, and a continuing period of data analysis. A second data collection period then commenced, in which paramedics were recruited through a theoretical sampling process based on the concepts that had begun to emerge. This theoretical sampling included 5 additional interviews (2 interviews with ECPs, 2 with frontline paramedic managers with dispatch experience and 1 with a paramedic educator), and four focus groups.

From the data, four key theoretical constructs arose that provide insight into the decision making process utilised by participants; role perception, education and training, operational demands, and confidence.

### Role perception

**Fig. 2:**
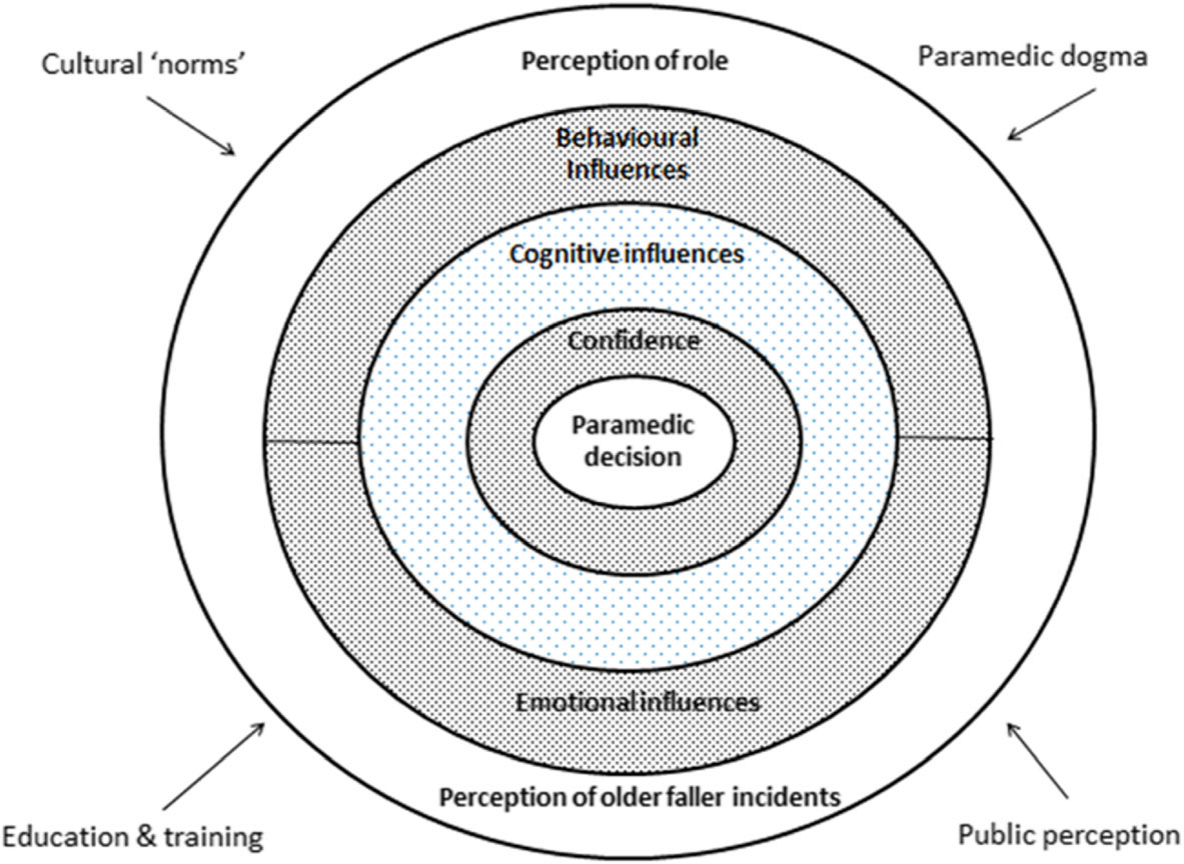
Decision making model of paramedic decision making when caring for older people who have fallen

Role perception was the dominant theoretical construct arising from the analysis. Paramedics experience confusion over their role and this has a substantial impact on the decision making process when caring for older fallers (Fig. 2).

Participants largely saw their role as delivering emergency, life-saving care to patients with “legitimate” health problems. There appears to be a cultural determination that high acuity work involving patients with potentially life threatening conditions is “legitimate” or “good work” or, as it was commonly described, “sexy work”. The cultural reality put forward by many participants, was that …“falls aren’t sexy.” [Regional ICP/Educator/21 years of service].

“I think the culture is…generally as ambulance practitioners, we like the blood and guts and gore and the glory jobs and the front page of the paper jobs and…the routine stuff where picking people up off the floor can be a little bit - it’s not glamorous, it’s not fun; there’s no adrenalin rush involved in it.”[Metro ICP/7 years]

Paramedics saw themselves as highly trained to manage patients with life-threatening conditions and it became evident that everything around them, culturally and organisationally, reinforces that this is what they are here to do. This is however at odds with the reality of ambulance service provision and the nature of the work they increasingly attend; “…if you think about lights and sirens, you don’t think about nanas who’ve fallen out of bed.” [Metro ICP/6 years]

“I think when I joined the job I was expecting a lot more real work…I've had a decent amount of real work but I just think that we do a lot of low-acuity work these days which is quite frustrating for me personally.” [Regional QP/7 years]

“We still see ourselves as primary, high acuity emergency responders. That’s what we want to be but the reality is we’re not anymore.” [Metro ICP/Educator/20 years]

As ‘paramedic role identity’ began to emerge as the core category, data collection began to incorporate material from websites, industry journals, news media and entertainment television. These sources, particularly the internal industry online sites and journals, portrayed paramedicine, through stories about major motor vehicle accidents, helicopter rescues, major traumatic injuries and resuscitation for cardiac arrest situations – the ‘sexy work’. A strong sense of ‘urgency’ and ‘emergency’ emanated from the written and visual data. Irrespective of the nature of the problem, the media reported almost without exception that the patient was “rushed” to hospital. This is again at odds with what the participants felt they were engaged in the majority of the time. Belief as to what the role of a paramedic is in turn influences the perception of how “legitimate” a particular case is in terms of being real work; this perception pervades the subsequent approach to a given case and the decision making process that follows.

There was a strong sense that cases involving older fallers did not constitute real paramedic work, and that these cases could prevent or inhibit attendance at real work should it occur; “… it’s [falls] the stuff we do…between real work” [Regional ICP/educator/20 years]. Falls were commonly described as type of work that was “not sexy”, perhaps reflecting the legitimacy or worthiness of such work within paramedic culture; “… people don’t come into the job to deal with nana falling” [Regional QP/educator/19 years]. Falls were consistently categorised as low acuity and were commonly described using words such as “frustrating”, “routine”, “annoying”, and “straight forward”.

“… paramedics love the exciting side of things and with a couple of television shows around now, Recruits, Paramedics, Triple 0 Emergency, that sort of stuff, All Saints, people see the exciting stuff and that sounds great and drive with the siren on and so on…I think a lot of people are drawn to the Ambulance Service because it’s – it can be exciting, and falls are – let’s face it, they’re not exciting. I find them interesting, but they’re not exciting.” [Metro ICP/frontline supervisor/24 years]

Paramedics, particularly ICPs, were frustrated that cases involving older fallers might make them unavailable for life-threatening, critical cases - ‘real paramedic work’; “…we’re supposed to be doing the acutely unwell patients…we want to be available for that stuff” [Metro ICP/9 years]. Contributing to this frustration was an altruistic and strongly held belief that high-acuity ‘real work’ is where they can make the most difference and do the most good.

Amongst the frustration arising from the frequency of attendances at incidents involving older fallers, a distinction between the ‘incident’ and the ‘patient’ was clear; paramedics described incidents involving older fallers in this way, but not the patients themselves. In fact, paramedics displayed a legitimate fondness towards the older patient who had fallen, and a sense of empathy was clear.

“… you can whinge to each other in the car but when you get there…you’ve just got to realise, it’s not the patient’s fault.” [Metro ICP/25 years]

A notable contrast became evident between QPs and ECPs. ECPs saw falls cases as legitimate work and generally spoke with no sense of frustration or annoyance when outlining experiences with such cases. ECPs, having undertaken additional advanced clinical education (a 380 h intensive course within the study jurisdiction) focussed on the assessment and management of low acuity conditions, minor injury and illness, chronic disease and geriatric medicine (including falls), did not appear to experience the mismatch of expectation to reality encountered by qualified paramedics. They may have been culturally reset during their specialist ECP training during which they have gained a new appreciation for cases involving older fallers and are more accepting of these as legitimate work; several described how their thinking had changed and that they felt better able to provide care to this population.

“… before it was easier to transport… didn’t have the tools or the background…or the access to the networks to…safely make the non-transport decision. After ECP, we’ve got…falls screening tools…more knowledge on musculoskeletal injuries, more anatomy and physiology to do better assessments” [Regional ECP/ICP/13 years].

Qualified paramedics described frustration with the frequency at which they encountered perceived low acuity work, and calls involving older fallers were acknowledged in this category. The confusion around paramedic role expectation becomes evident again; paramedics who are culturally and organisationally “indoctrinated” to see themselves as providers of emergency and high acuity care find themselves spending the vast majority of their shift responding to low acuity incidents, creating a substantial and powerful perception-reality mismatch. This manifests in negative feelings towards low acuity work – paramedics felt “frustrated” and ‘tired” of attending a high volume of low acuity incidents that did not involve life threatening, emergency work. Several paramedics described this phenomenon as getting “burnt out” on low acuity work – theoretically we have labelled this ‘low acuity fatigue’. Repeated attendance to older fallers creates complacency and a misplaced preconception before arriving at the scene that there will be nothing seriously wrong with the patient. The paramedic who perceived falls cases in such a light was at risk of making a poor decision regarding how to manage the case, leading to a cursory examination, poor information gathering, and sub-optimal clinical decisions;

“…I think it’s also the fact that we deal with so much low acuity work that you just stop thinking” [Metro QP/8 years].

One paramedic likened the consequences of low acuity fatigue to the story of the boy who cried wolf; attendances at low acuity cases had become so common and there was so often nothing serious identified, that they became complacent.

“… the routine callers are the ones that you're going to lose your… job over or your reputation over…you'd go to the job with a pre-conceived notion. This is old so-and-so; they're always falling out of bed. Let's get them back to bed, they'll be fine” [Metro/ICP/ECP/20 years]

The perception of whether falls cases constitute ‘real work’ forms the framework for the approach to decision making when attending the patient (Fig. 1). Paramedics who felt falls cases were ‘real work’ most commonly described a hypothetico-deductive approach to decision making, particularly the ECPs. These paramedics were able to find a clinical challenge at falls cases;

“I like…the detective process of going in and figuring out if your patient is well enough to leave at home or if they’re sick, how sick are they?” [Metro ICP/6 years]

In contrast, those who felt falls cases were not ‘real work’ appeared to use a more pattern-based decision making approach, anchored in the unqualified experience of past cases and heuristics, and subject to cultural norms and expectations which position falls as simple, straightforward, uncomplicated and low-acuity;

“… they’ll [the paramedic] sometimes just walk in with a blood pressure cuff…they’ve made a decision before they even walked in the door.”[Metro QP/5 years]

### Education and training

The paramedics’ capacity to make good decisions is limited in part by the quality of education and training they receive. Paramedics reported feeling ill-prepared to manage cases involving older fallers, believing they received little education or training in this area. This perception was supported by paramedic educators who acknowledged that falls-related education and decision making training was insufficient. As paramedics described their patient assessment processes and priorities, they were predominantly focussed on looking for and fixing injury, or as one paramedic said; “looking for blood on the floor” [Regional ICP/educator/24 years]. Aside from the ECPs, few paramedics described assessment of falls risk factors or falls risk, or investigation of medical causes when discussing decision making processes. Some acknowledged that in an ideal situation they would do this, but given they have had insufficient or no training for such assessment, they saw no point in doing it if they could not do it properly. The fact that paramedics appear to receive little training in management of older fallers might be seen to be an implicit, inadvertent message from the organisation that affirms the perception that this type of work is not real work, that ‘this is not your role’;

“… if this was really our job, they’d train us properly to do it” [Metro QP/6 years].

Some paramedics, particularly those with education experience, reflected on the imbalance between the amount of education and training committed to infrequent procedures for life-threatening conditions and that committed to low acuity cases such as falls; the frequent classification of all falls cases as low acuity was prevalent, despite the reality that such incidents can present anywhere on a continuum of acuity and severity.

A contradiction was evident regarding the perceived inadequacy of training and the desire for better education and support around falls, and the lack of acceptance of an algorithmic falls protocol (known internally as ‘T19’) introduced to support decision making. Despite having been introduced more than two years previously, paramedics were generally unfamiliar with this protocol, acknowledging that it played little or no role in their decisions. When asked about their understanding of the protocol, one paramedic commented ‘… we have one, do we?’ [Regional QP/10 years]

“T19 … that’s falls in the elderly, yeah? Glossed over that one in the back of the book…it’s not looked at, it’s not a fancy, fun, exciting protocol, it’s not one that we get tested on routinely.” [Metro ICP/6 years]

“So the time I spend reviewing my protocols was on, you know, time critical, life threatening stuff and…I actually didn’t open the book to T19” [Metro/ICP/Frontline supervisor/24 years].

Those who were aware of the T19 protocol demonstrated a reluctance to use an algorithmic approach to decision making, suggesting that such an approach was too constrictive to paramedic practice.

“… you can’t just walk in with the protocol book…you look at some of these algorithms and it can be quite complicated. They’re good if I’m doing an exam tomorrow and I’ll have to sit there and read over it, but outside of that?” [Metro QP/6 years]

Several participants commented that the release of the falls protocol was not accompanied by any underpinning knowledge, training or promotion during formal training workshops or clinical update sessions;

“… must have slipped off the table, that one” [Regional QP/17 years].

Though the participants believed that supporting education and promotion of the falls protocol on its release was insufficient, they had not themselves sought to develop a familiarity with it or take responsibility for understanding it. In contrast, participants admitted they would learn new protocols for life-threatening conditions “off by heart” even if they were not well supported by in-service education and professional development.

While paramedics agreed they were poorly trained and lamented the lack of education they received about falls, many felt that engagement in fall risk assessment or injury prevention initiatives was beyond what a paramedic should be doing.

“Really, our job is to…make sure they’ve got a pulse when they get to the ED (emergency department). All the rest of it is nice stuff to do, but I think our job in the system is that.” [Regional QP/20 years]

Comparison of data between vocationally trained paramedics and those with higher education degrees revealed no differences in perceived preparedness to manage older fallers. However, further analysis of the effect of education on practice was limited by the heterogeneous nature of paramedic education and the inability to control for other variables affecting decision making.

There was concern that spending prolonged periods of time on scene with an older faller engaging in such assessment would prevent them from responding to life-threatening work that they felt obliged to be available for. Also, there was a strong belief that even if paramedics received better training in falls management and fall risk assessment, many paramedics would “not bother to do it anyway.” [Metro ICP/9 years].

### Operational demands

Intrinsic and extrinsic pressures arising from the employer’s need to manage substantial emergency ambulance demand impact substantially on paramedics’ decision making processes when providing clinical care to older people who have fallen. Operational demands influenced decision making in two ways.

First, paramedics felt a constant pressure to be available to respond to real work, that is, the life-threatening high acuity work. This pressure can be cumulative, intensifying as they are repeatedly dispatched to perceived low acuity cases such as falls, rendering them unable to respond to what they perceive they should be doing. In the context of falls, this can manifest in shortcut assessments that provide limited opportunity for holistic assessment of an older faller. The source of the pressure lies both within the paramedics themselves as they are subject to organisational norms and beliefs, and from the dispatch centre and the organisation through behaviour and performance measure indicators that promote “restrictive” scene times with no perceived consideration for the clinical needs of the patient. Several participants reported frustration with “welfare checks” issued over the radio by the dispatch centre when a crew’s time on scene approaches twenty minutes; paramedics interpret these checks as “hurry ups”, a subtle suggestion to “get along with it.”

“Are you going to transport? Can you clear? Are you going to transport? Are you going to transport? You’re sort of making these - oh well, we’ll pop them back in the chair and we’ll clear and we’ll do whatever it is they’re wanting us to do which, inevitably, ends up not being anything that particularly important anyhow.” [Metro ICP/6 years]

“I think you have to come to the point where you need to realise that the patient you’ve got is the patient you’re responsible for, regardless of what’s happening around you and the external pressures of operations.” [Metro QP/8 years]

Second, operational demand is intrinsically linked to hospital delays, which deplete frontline emergency ambulances and decrease the ability of the service to respond to operational demand. Hospital delays impact heavily on paramedic decision making by placing operational concerns above clinical considerations, particularly in regard to the transport decision; “… the decision isn’t being based on the patient that they see before them, the decision is being based on the external pressures that are being brought to bear.”

“If you know that you’re going to have an immediate off-load and your turn-around time will be less than an hour, in my opinion, you’re much more likely to transport…if you know that you’re going to be stuck for three hours, you’re going to be encouraging this patient to go down other pathways…but it might not be as safe, might not be as risk averse as the ambulance service would like.” [Metro ICP/7 years]

“… do non-transports go up when we’ve got extensive hospital delays? Absolutely. Are they sometimes not the most sound decisions? Absolutely.” [Metro ICP/Frontline supervisor/17 years]

Operational pressures were felt most noticeably by intensive care paramedics (ICPs), who were the clinical group most conflicted with regard to perception of their role. ICPs spoke passionately about the need to be available for serious work at which they feel they can have the most impact or to provide backup to more junior paramedics in need of clinical support. ICPs were frustrated about attending cases involving older fallers, as they saw these cases as a diversion from the work they should be doing.

### Confidence

Decision making by paramedics when caring for older fallers is strongly influenced by a pervading fear of not being supported by their organisation if an adverse event were to arise secondary to their decision making; this was described as “being shafted”, a phrase that featured prominently in the data.

“I think I…assess patients very well…you know, I’m 99.9% sure that they’re going to be fine but it’s just that, well, what if the ambulance service doesn’t look after me and I lose my job?” [Metro QP/5 years]

The overwhelming sentiment among participants was one of cynicism toward their organisation, particularly towards what were described as “middle-level operational” managers, and how adverse events are handled. Contextually this was mostly in relation to non-transported patients, a decision which many were uncomfortable making due to the perception that they are opening themselves up to risk of organisational reprisal; “… until the ambulance service will back my decision of leaving that patient at home, I’m not confident to do it regardless of what protocol comes in” [Metro ICP/10 years].

This lack of confidence fuelled a strong culture of “cover your arse” in which decisions were frequently made based on what is best for the paramedic; ‘paramedic-centred’ decision making. Patients who may have been suitable for non-ED care were transported out of a sense of self-protection for the paramedic, not through a fear of the patient not being safe.

“So, um, covering our arses is a big deal…It’s a massive culture within the ambulance service” [Metro QP/8 years]

Those with little confidence in the organisation spoke of transporting all older fallers irrespective of the needs of the patient, therefore eliminating risk of being “hung out to dry”. This approach was described as the “you call, we haul” approach to managing the transport decision; this approach removes the need to make a decision. There was also a sense that some paramedics lacked confidence in their own clinical ability to manage an older faller; they are not well trained in managing older fallers and so may feel a sense of helplessness when confronted with these patients, resulting in a default position of transport in the absence of being capable of doing anything else.

“… when it comes to leaving them at home, if it was to come back to me, I don’t have the right level of training or knowledge to justify myself” [Metro QP/ 5 years].

In contrast, paramedics who stated they felt confident in their clinical ability relative to this population predominantly spoke of doing what is best for the patient, and if that meant not transporting to ED, then that is what they would do; “… I’m not going to sit there and say oh no, you really must be going to see the doctor today because I need to cover my arse” [metro QP/8 years]. These participants also expressed cynicism toward the organisation, but their confidence as clinicians and decision makers over-rode those concerns. A more ‘patient-centred’ approach to decision making was evident among this group who, when discussing non-transport, appeared to be primarily concerned about the patient being safe, not about themselves being safe from the organisation (‘paramedic-centred’ decision making).

## Discussion

In this study we have explored decision making by paramedics when providing care to older people who have fallen, presenting a perspective on decision making used in this context. The original proposal for the study focussed largely on the decision making process leading to whether an older faller is transported to hospital following paramedic assessment. True to the inductive nature of grounded theory methodology, the participants, and subsequently the data, led us away from the transport decision being the focal point of the study. As a result, a wider lens was used which led to broader exploration of how paramedics make decisions in this context, though the non-transport decision remained prominent throughout.

At the heart of our theoretical model explaining paramedic decision making when caring for older fallers is the core theoretical construct of *role perception* (Fig. 1). Participants saw falls cases as low acuity work that was not what their ‘core business’ was supposed to be. There is an emotional conflict arising from the perceived imbalance in the work they have been trained to do and the work they actually spend their time doing, and is very likely to be broader than older fallers and include all perceived low acuity work. This finding has similarities with a small Canadian study that reported conflict in role perception in the context of paramedic attitudes towards low acuity incidents more broadly [26]. Interviews with 13 paramedics revealed cynicism and disengagement toward low acuity work, emotions that were adopted as a coping mechanism to overcome the frustration arising from constant immersion in perceived low acuity work. The Canadian and Australian ambulance services systems have many similarities in case-mix and scope of practice, which might explain the similar findings; however, the university pre-employment training model seen in Australia does limit the applicability of these finding to the Canada which has yet to evolve to such an education model.

The recent emergence of paramedicine as a recognised health profession [27] may explain the absence of exploration into role identity and its impact on practice; paramedicine is currently in a stage of adolescence – knowing what it does and does not like, knowing what others want it to be, but not really knowing what it wants or needs to be. Regarding incidents involving falls in the elderly, a clear stream of organisational communication that consciously and explicitly position falls and care of older people as important work (‘real work’), supported overtly through education and training and accuracy in role portrayal, may help in establishing these cases as ‘legitimate’ work in paramedic culture. There are workforce implications arising from these results, too. Recruitment and marketing strategies targeting future paramedics should ensure prospective employees are aware of the role of a contemporary paramedic and post-employment induction, education and training should include concerted efforts to dispel myths and misconceptions relating to the nature of paramedic work. This may contribute to less confusion regarding the role of a paramedic, increased job satisfaction, reduction in early career ‘burnout’, and improved staff retention.

In this study, paramedics’ individual beliefs as to what their role should be influenced their attitude toward cases involving older fallers, reinforced by cultural norms and conformity to peer perceptions of what is real paramedic work. Their intent, or belief in their ability to manage older fallers, appeared low due in large part to insufficient knowledge and education. The influence of social norms is particularly relevant given the model of care practiced in this ambulance service; there are two paramedics on almost every ambulance, both of whom are present and formally responsible for decisions that are made in relation to patient care. One paramedic tends to take the lead role at each case, with the other taking a more subordinate role in the patient encounter. They may switch between these roles throughout the shift, sharing the ‘burden’ of patient care. That a perception-based decision can be made, as proposed in this study, suggests that with two paramedics present, they either both have the same perception and attitude, or the attitude of one is influenced by the perceived attitude of the other (the social norm). This interplay between two qualified paramedics, the division of clinical accountability, and the tendency of the paramedic in the subordinate role to conform with what they may perceive to be an expected and accepted decision making approach to particular patient group might be significant in understanding the process and therefore requires further investigation.

We found that paramedic attitudes toward elderly falls cases and the cultural and organisational subjective norms which fuel paramedics’ perception of role identity, are inextricably linked and in this population of paramedics, were strong determinants of an individual paramedic’s decision making. This finding is consistent with several previous studies investigating paramedic behaviour in the context of decision making. Weber et al. investigated paramedic ‘behaviour’ when making decisions in relation to administration of morphine by a population of Australian paramedics [13], using Ajzen’s Theory of Planned Behaviour (TPB) model as a theoretical framework [28, 29]. TPB seeks to explain human behaviour as being the result of a person’s attitude (their self-beliefs), social norms (perceived beliefs of peers, co-workers, or social group) and their intent (their belief in their ability to be successful in the behaviour of interest). The authors identified subjective norms as being the strongest predictor of decision making behaviour, postulating that conforming to perceived cultural norms and feeling accepted by peers is a key driver of that behaviour. In a similar study, Bannerjee et al. explored decision making behaviour in the context of performance of intravenous cannulation in a group of UK paramedics [16]. Using Ajzen and Fishbein’s Theory of Reasoned Action as their framework [30], they found that paramedic ‘attitude’ was the key predictor of subsequent behaviour regarding performance of cannulation, more so than subjective norms in their population. Both studies concluded that addressing attitudes was the key to changing paramedic decision making behaviour.

Emotional and cognitive influences (termed ‘affective dispositions to respond’ and cognitive dispositions to respond’, respectively) were prominent in the decision making process, both of which have been clearly shown to impact on rational clinical reasoning processes [31]. Among participants in this study, being dispatched to a case involving an older faller was associated with predominantly negative feelings and emotions such as frustration, disappointment, indifference and, for some, anger. These feelings arose directly from the previously described conflict in role perception and work expectation. The presence of these emotions appears to induce a lowered state of cognitive arousal while driving to the incident and during the subsequent interaction with, and assessment of, the patient. This emotional dysregulation compromises the degree of ‘cognitive control’ [32] the paramedic has during their reasoning and decision making, increasing the risk of errors affecting judgements and clinical decisions. The cumulative effect appears to be decision making dominated by heuristic thinking strategies that compromise the integrity of the reasoning process and impact on patient safety. Heuristics, or cognitive shortcuts, can represent a valid, useful, and economical approach to decision making when used in the right situation in the hands of an experienced clinician [33]. However when heuristic thinking processes arise secondary to emotional dysregulation in a clinician with limited meta-cognitive capacity and cognitive control, patient care can be become sub-optimal.

There is no literature discussing meta-cognition in the paramedical context. We found evidence in this study to support a hypothesis that paramedics may not be culturally or educationally aligned to the concept of metacognition, or ‘thinking about how they think’, illustrated by one participant who, when asked to describe how they think when making decisions, simply stated…“I don’t know how I think!” [Metro ICP/6 years]

Cognitive dispositions to respond (CDR) featured prominently in the decision making process of paramedics in this analysis, most notably anchoring, ascertainment bias, overconfidence bias, and posterior probability error. Teaching metacognition to paramedics, training them to think about how they think and to consciously reflect on how they make decisions may increase awareness of affective and cognitive dispositions to respond on an individual and cultural level and promote enhanced clinical decision making [34]. There is evidence that such training improves clinical reasoning in junior physicians [33, 35] but further research is required to explore if this evidence may be generalisable to paramedics. Education in cognitive strategies for reducing reasoning bias, and a clearer professional perception of who they are and what their role is, may decrease the tendency toward heuristic, non-analytical decision making seen in this study.

The construct of *confidence*, in the context of a lack of faith that their employer will support them in the event of an adverse outcome, is consistent with several earlier studies in which paramedics identified the perceived need to ‘cover your arse’[36–38]. In Halter’s UK study exploring decision making by paramedics when attending older fallers, ‘professional protection’ was a key theme that emerged, weighing heavily on paramedics when determining whether to transport or not [11]. Similarly, Porter et al., in a qualitative study from the UK involving three focus groups, identified a lack of faith among paramedics that their organisation would support the decisions they make regarding non-transport [37]. This lack of faith promoted a ‘cover your arse’ mentality, particularly with regard to clinical documentation. Despite this lack of confidence in the organisation backing their decisions, paramedics seem unreceptive to initiatives to support paramedic decision making, such as the introduction of a ‘falls protocol’ as described in this study. The lack of acceptance and utilisation of the algorithmic tool seen in our study mirrors experiences in the UK when a similar tool to help arrive at a transport decision for older fallers was introduced [11]. The ambulance systems of Australia and UK, particularly with regard to organisational culture, education and case-mix are quite similar in many ways, hence the findings of our present study are more than likely quite generalizable to the UK.

It is possible that it is the style of support (clinical algorithm) as opposed to the concept of support that may be unsuitable to paramedic culture and practice. The paramedics in this study described a reluctance to use an algorithmic protocol when managing cases involving older fallers, believing it was impractical and stifled the judgement-based decision making they should be engaging in. The role of algorithmic protocols in paramedic practice is an area that is in need of research, as there is a tendency to ‘protocolise’, possibly as a governance and patient safety measure under the belief that if it is protocolised then paramedics will comply.

These findings have implications for policy in professional bodies, ambulance services and undergraduate higher education paramedicine programs. Implications for ambulance services have been alluded to elsewhere in the paper, but revolve largely around working to ensure that all aspects of an organisation consciously work to instil organisational value on lower acuity work, particularly that involving older fallers. This requires a multi-faceted and consistent approach, that involves educational, operational, clinical and media initiatives to explicitly and implicitly position non-life threatening work as ‘real paramedic work’. For higher education providers, there are implications for curriculum design and teaching methods, in that these should embed non-life threatening, low acuity content systematically and overtly throughout a curriculum rather than focus largely on higher acuity cases as source material for learning activities. Further, these providers should consider the ‘hidden high acuity curriculum’ that commonly exists, perpetuated by unintentional focus on higher end clinical work through choices in imagery, promotion, media and educator storytelling. With university level education training now mandatory in Australia, this region is perfectly positioned to provide student paramedics with broader impressions of healthcare and the role that ambulance services play within it. Finally there are implications for the profession more broadly, often meditated by high profile professional bodies representing the Australasian workforce. These findings suggest that there is work to be done within these organisations and the messages the convey, similar to what was described earlier for ambulance organisations. External public perceptions of what paramedics do may be as influential in forming paramedics’ perception of what they do and what real work is, as internal factors previously described. Professional bodies may be perfectly positioned to challenge misconceptions surrounding contemporary paramedicine in the public domain, and adopting a more aggressive approach to managing community and paramedic expectation of what paramedics do could yield benefits.

Falls in the elderly are a prominent feature in the emergency workload of contemporary paramedicine systems, and will only become more so in the coming decades as the population ages. This study has illuminated the area of paramedic decision making in relation to this growing population of patients, and identified several key areas deserving of closer investigation. We have proposed a theory explaining paramedic decision making in the context of older fallers (Fig. 1), one that may have validity in other contexts involving patient groups presenting with perceived low acuity problems. We have generated several hypotheses and future directions for research that if considered by paramedics, educators of paramedics and employers of paramedics, could contribute to improving the care of older fallers, optimising the care of less urgent, lower acuity cases more broadly and may potentially improve paramedic job satisfactions.

## Limitations

There are limitations that should be borne in mind when considering the results of the study. First, the study was conducted within a single ambulance service hence the findings may not be generalisable to other populations of paramedics in other ambulance services. It must be emphasised though that such a lack of generalisability is not a limitation per se as it might be considered in quantitative research, but rather a feature of the epistemological underpinnings of the constructivist stream of grounded theory, and qualitative research more broadly. A constructivist paradigm is founded on the belief that there is no single, objective truth, but rather that knowledge is a co-creation between researcher and participants that would therefore be different if conducted by other researchers at another time in another ambulance service. Therefore the results of this study represent an interpretation of the stories, experiences and knowledge elicited by the researcher, and put forward by the participants.

Second, the data analysis and coding was performed by a single researcher who was also an experienced paramedic. This could give rise to concerns regarding potential for bias, however again it must be recognised that the study was conducted using an interpretive methodology and that the researcher is inherently involved in constructing the results. The paramedical experience of the primary researcher was transparently reported as was the process of reflexivity that was exercised throughout. The emerging knowledge was transparently reported and shared amongst the research team, accompanied by ‘memos’ acknowledging personal feelings and experiences. Using these methods, the subjectivity of the analysis was regularly checked and challenged by the broader research team.

It must also be kept in mind that this was a single centre analysis, focussing on a single ambulance service within an Australasian paramedicine context. While generalisability across Australian ambulance services is reasonable, consideration should be given to variations in international health systems and paramedic services. For example, the mandatory higher education qualification now existing in Australian ambulance services sets it apart in entry level training from most countries. Given the emergence of ‘education and training’ as a key construct in this research, generalising these findings internationally should be done with caution. The findings relating to the key theoretical construct of ‘role identity’ are more likely to be generalizable as many developed countries are experiencing changes in healthcare utilisation patterns and an ageing population comparable to Australia.

Finally, this study only looked at decision making from the paramedics’ perspective. Patients and their families or carers were not involved in data collection – this was a conscious decision, as we were specifically interested in explaining the process from the paramedic perspective. Exploring the patient and family perspective on decision making, and the role they perceive themselves to play in that process, represents an important avenue for future research that would serve to further our understanding around paramedic decision making in the out-of–hospital setting.

## Conclusion

Paramedic decision making, when providing care to older people who have fallen, is profoundly influenced by perception of role and the cultural and organisational constructs of what is real work for paramedics. Perceived inadequacy of preparedness to provide care to older fallers, characterised by insufficient education and training, a lack of confidence, and constant pressures arising from management of operational demand, inhibit the provision of patient-centred, carefully considered care to this population.
